# Weight and Lipid Levels in People Living With HIV and Initiating a Dolutegravir-Based Regimen in a Resource Limited Setting: A Prospective Study

**DOI:** 10.1155/2024/4620951

**Published:** 2024-11-14

**Authors:** Rudo Chakanetsa, Raylton P. Chikwati, Itai Chitungo, Cuthbert Musarurwa, Donald M. Tanyanyiwa, Justen Manasa, Vinie Kouamou

**Affiliations:** ^1^Unit of Chemical Pathology, Department of Laboratory Diagnostics and Investigative Sciences, Faculty of Medicine and Health Sciences, University of Zimbabwe, Harare, Zimbabwe; ^2^Department of Chemical Pathology, Faculty of Health Sciences, University of the Witwatersrand, Johannesburg, South Africa; ^3^Sydney Brenner Institute for Molecular Bioscience, Faculty of Health Sciences, University of the Witwatersrand, Johannesburg, South Africa; ^4^Department of Biomedical Laboratory Sciences, School of Health Sciences, College of Medicine and Health Sciences, University of Rwanda, Kigali, Rwanda; ^5^Department of Chemical Pathology and Cytogenetics, Sefako Makgatho Health Sciences University, National Health Laboratory Service, Dr George Mukhari Academic Hospital, Pretoria, South Africa; ^6^Department of Internal Medicine, Faculty of Medicine and Health Sciences, University of Zimbabwe, Harare, Zimbabwe; ^7^Biomedical Research and Training Institute, Harare, Zimbabwe

**Keywords:** BMI, body weight, dolutegravir, HIV, lipid levels, Zimbabwe

## Abstract

**Background:** Following the 2018 World Health Organization's (WHO) guidelines on HIV treatment and management, the Zimbabwean government has embraced dolutegravir (DTG)-based regimens as the preferred first-line treatment for people living with HIV (PLWH). Despite this implementation, there remains a paucity of knowledge on the potential associations between DTG-based regimens, body weight and blood lipid levels among PLWH in Zimbabwe. Thus, the aim of this study was to investigate variances in body weight and blood lipid levels at two distinct timepoints—baseline and 6-month post-DTG initiation.

**Methods:** We conducted this study between November 2021 and April 2023 among ART-naïve individuals initiating a DTG-based regimen. Participants were recruited from a tertiary clinic in Zimbabwe. Body weight, standing height and blood lipid levels were measured at baseline and 6-month post-DTG. Changes in weight, body mass index (BMI) and lipids levels were assessed using the paired Student's *t*-test and Wilcoxon signed rank test. Multivariable logistic and linear regression analysis was used to assess risk factors associated with changes in weight, BMI and lipid levels.

**Results:** A cohort comprising 130 study participants, characterised by a mean (±SD) age of 40.0 (±11.0) years at baseline, was subjected to a 6-month regimen of DTG-based therapy. The outcomes revealed statistically significant alterations in various physiological parameters. Specifically, post the DTG-based intervention, there were substantial increases observed in body weight (*p* < 0.001), BMI (*p*=0.003), total cholesterol (TC) levels (*p*=0.002) and high-density lipoprotein cholesterol (HDL-C) levels (*p* < 0.001) in comparison to their baseline values. Intriguingly, the corresponding triglyceride (TG) levels exhibited a noteworthy decrease (*p* < 0.001). Notably, individuals aged 40 years or older exhibited a positive association (*p*=0.022) with increased TC levels and concurrent weight gain. Furthermore, current employment emerged as another factor positively linked to increased TC levels and weight gain.

**Conclusions:** Upon the initiation of DTG, discernible elevations were observed in body weight, BMI and lipid levels. This study represents the first comprehensive assessment of lipid profiles and weight gain among this population in Zimbabwe, filling a critical gap in the existing literature. These findings, while indicative of short-term effects, underscore the imperative for further investigative efforts aimed at elucidating the prolonged consequences associated with DTG-induced weight gain and increased lipid levels and its underlying mechanisms.

## 1. Background

Improved access to antiretroviral therapy (ART) has been associated with decreased mortality in people living with HIV (PLWH) due to its effect on viral replication and immune restoration [1]. However, the increased longevity of PLWH has also been associated with increased noncommunicable diseases that include Type 2 diabetes mellitus and cardiovascular diseases [1, 2]. According to the 2021 Zimbabwe Population-based HIV Impact Assessment (ZIMPHIA) report, the prevalence and incidence of HIV among Zimbabweans aged between 15 and 49 years remain high, approximately 12.9% and 0.38%, respectively [3]. Following the 2018 World Health Organization (WHO) guidelines on HIV treatment and management [4], Zimbabwe has since embraced dolutegravir (DTG)-based regimens as the preferred first-line treatment for PLWH [5]. The adoption of DTG-based regimens has become a cornerstone in resource-limited settings, particularly in the pursuit of effective and accessible ART [6, 7].

However, side effects associated with DTG have recently been documented. These side effects include lipodystrophy and insulin resistance among others [8–10]. Clinical outcomes in patients on DTG-based ART are being monitored in real world and programmatic settings [6]. Despite extensive monitoring efforts, a noteworthy gap persists in the literature concerning investigations on alterations in body weight and blood lipid profiles among PLWH following DTG-based ART in the specific context of Zimbabwe.

We therefore assessed differences in body weight and blood lipid levels at two timepoints, baseline and 6-month post-DTG initiation in the Zimbabwean population.

## 2. Materials and Methods

### 2.1. Study Design, Population and Setting

This prospective cohort study was conducted between November 2021 and April 2023. Consenting participants were ART-naïve adults (aged ≥ 18 years) initiating on a DTG-based regimen or re-initiating ART after defaulting for at least 3 months. All participants received the single-dose tablet of tenofovir disoproxil fumarate/lamivudine and DTG (50 mg). Participants were seeking care at the Parirenyatwa Hospital Family Care Centre (PHFCC), a tertiary level clinic in Harare, Zimbabwe.

### 2.2. Study Procedure

Individuals were consecutively invited to participate in the study and screened for eligibility. Socio-demographic and clinical data (sex, age, marital status, employment status, highest level of education attained, weight and height) and history of ART exposure were obtained through an interview-based questionnaire and standardised measurements. At enrolment and after 6 months, all participants were attended to by clinic physicians for routine clinical assessments as per the national treatment guidelines. On both occasions (enrolment and 6 months follow-up), 5 mL of whole blood were collected into ethylenediaminetetraacetic acid (EDTA) tubes for quantifying lipid levels. Participants weight and height were also measured at both time points.

### 2.3. Laboratory Methods

The collected blood specimens were centrifuged at 3000 revolutions per minute (rpm) for 15 min to separate blood plasma. The plasma was then stored at −80°C at the Infectious Diseases Research Laboratory, University of Zimbabwe. Prior to lipid measurements, specimens were retrieved and thawed only once at room temperature for 30 min. Lipid levels were then measured on the BS220 Mindray chemistry analyser (Shenzhen Mindray Bio-Medical Electronics Co., Ltd, Shenzhen, China). All analyses were performed in accordance with the manufacturer's instructions and principles of good clinical laboratory practice. The measurement of lipid levels was based on the combination of enzymatic and colorimetric methods on the individual plasma lipids. The measured plasma lipids comprised of HDL-C, TC and TG. The measuring range and coefficient of variation for HDL-C, TC and TG were 0.03–4.66 mmol/L, 0.52%; 0.08–19.4 mmol/L, 0.61% and 0.02–11.3 mmol/L, 0.80%, respectively. Low-density lipoprotein cholesterol (LDL-C) was calculated using the Martin Hopkins equation [11]. Body mass index (BMI) was calculated by dividing the study participant's weight (kilograms) by the square of their height (metres).

### 2.4. Diagnostic Criteria for Dyslipidemia

Dyslipidemia was defined as per the National Cholesterol Education Programs, Adult Treatment Panel III (NCEP ATP III) guidelines as follows: elevated TC- (TC > 5.18 mmol/L), elevated LDL-C (LDL-C > 2.59 mmol/L), decreased HDL (HDL-C < 1.04 mmol/L) and elevated TG (TG > 2.26 mmol/L) [12]. Participants were considered as having dyslipidemia if they met any one of these criteria.

### 2.5. Statistical Analyses

All data analyses were performed using the Stata 17 software (StataCorp LLC, College Station, TX, USA). Data visualisations were performed using RStudio 4.0 (RStudio Team, PBC, Boston, MA, USA). Descriptive statistics were used to summarise data. Nonparametric data were summarised as median or interquartile range (IQR) and parametric data as mean ± standard deviation (SD). Categorical data were presented as sample size and percentages (n,%). The Shapiro–Wilk test was used to assess the normality of continuous data. The Fisher's exact and Chi-square tests were used to compare differences across categorical data as appropriate. The Wilcoxon signed rank sum and Student's *t*-test for paired samples were used to explore differences between study variables at baseline and after 6 month of ART. Survivorship analyses comparing the baseline study variables between participants that were retained and lost to follow-up were also performed. The threshold for statistical significance was set at *p* value < 0.05. Univariable and multivariable logistic regression analysis were performed to explore associations between baseline variables (age, sex, baseline CD4, marital status and employment) and weight gain while on DTG. Participants' characteristics with *p* value < 0.20 in the univariable analyses were included in the multivariable logistic regression analysis. Furthermore, the dynamics of change in weight and lipid levels were investigated using univariate and logistic regression analyses. The rate of change in weight and lipid levels were the dependent variables and the same baseline variables assessed in the logistic regression models were used as independent variables. The threshold for collinearity in both linear and logistic regression models was set at a variance inflation factor (VIF) of less than 5.

## 3. Results

### 3.1. Baseline Characteristics of the Participants

In this study, we recruited 130 participants, of which 74 (57%) were females. The mean (SD) age of the participants was 40 (± 11) years, and the median (IQR) baseline BMI was 22.0 (19.2–26.0) kg/m^2^. Females had higher median BMI than males (23.2 (20.4–26.9) versus 20.3 (18.5–23.5) kg/m^2^, *p*=0.003). The majority of participants in the study were immunocompromised, characterised by a median (IQR) CD4 cell count of 196 (98–357) cells/mm^3^ (Table 1). A significant proportion of participants were currently married (72, 55.4%), and a substantial portion had achieved a secondary level of education (107, 82.3%). More than half of the study participants (69, 53%), were unemployed. At recruitment, many of the participants were ART naïve (107, 82%) and 18% had previously defaulted on ART (*n* = 23) (Supporting Information Table 1). ART-naïve participants had significantly lower CD4 cell count compared to those who previously defaulted ART (169 (74–351) versus 275 (198–436), *p*=0.04). Interestingly, a lower proportion of married individuals were found among previously defaulters compared to ART-naïve individuals (8 (34.78) versus 64 (59.81), *p*=0.03). No differences were found in age, weight or lipid levels between individuals who were ART naïve and those who had previously defaulted (Supporting Information Table 1).

### 3.2. Changes in Body Weight and Lipid Levels After 6 months on DTG

Out of the initial 130 participants enroled in the study, 80.2% (*n* = 105 individuals) successfully completed the 6-month follow-up assessment. Loss to follow-up was attributed to mortality in (7, 5.4%), as well as logistical and personal reasons. When comparing the baseline characteristics of participants retained and those lost to follow-up, no significant differences were found (Supporting Information Table 2). In participants who were followed up, the average weight gain was 5.8 kg (*p*=0.001), with a mean (SD) weight at follow-up of 67.6 kg (13.1) compared to 61.8 (11.5) kg at baseline. Of the 98/105 participants with follow-up data on weight, 42 (43%) experienced weight gain.  Table 2 illustrates that post-DTG initiation, participants exhibited significant alterations in weight (*p*=0.001), BMI (*p*=0.005), TC levels (*p*=0.003), and HDL-C levels (*p* < 0.001), all showing an increase. Conversely, TG levels demonstrated a significant decrease (*p* < 0.001) from baseline, while no significant changes were observed in LDL-C levels during the follow-up period (*p*=0.171).

The cut-off used for multivariable logistic regression analysis of on-treatment mean weight gain was at 5.8 kg. In the evaluation of risk factors encompassing age, sex, baseline CD4 cell count, marital and employment status through both univariate and multivariable logistic regression analyses, only current employment was associated with weight gain. Employed participants were approximately 4 times more likely to gain weight compared to unemployed participants (OR = 3.69, 95% CI 1.46–9.32, *p*=0.006) (Table 3). We found a significant difference in mean weight (6.2 kg, *p*=0.020) between employed and unemployed at follow up, with employed having a mean of 71.2 kgs compared to 65.0 kgs seen among unemployed despite the same baseline mean weight (*p* > 0.05). These findings demonstrate the contribution of employment in weight gain within this population.

Upon evaluating the same set of risk factors, encompassing baseline BMI, as predictors for observed alterations in TC, HDL-C and LDL-C, older age emerged as a significant predictor associated with an increase in TC concentrations. Participants of older age exhibited a notable elevation in TC concentrations, with an OR of 3.37 (95% CI 1.20–9.50, *p*=0.022). There was no association between an increase in TC concentration and sex, (*p*=0.055) (Supporting Information Tables 3, 4 and 5).

In a subanalysis shown in Supporting Information Tables 6, 7, 8 and 10, we assessed the relationship between the rate of change in weight and lipid levels over the follow-up period. Individuals with baseline CD4 levels ≥ 200 cells/mm^3^ experienced a significantly greater weight increase compared to those with a CD4 count < 200 cells/mm^3^ (Supporting Information Table 6). The estimated effect was a 0.82-unit increase in weight change and was statistically significant (*p*=0.002). Individuals older than 40 years had significantly greater increase in total cholesterol and LDL-cholesterol levels compared to those younger than 40 years (Supporting Information Tables 7 and 8). The analyses also showed no association between differences in sex, marital status, employment status, triglycerides and HDL-cholesterol (Supporting Information Tables 9 and 10).

### 3.3. Dyslipidemia

**Figure 1:**
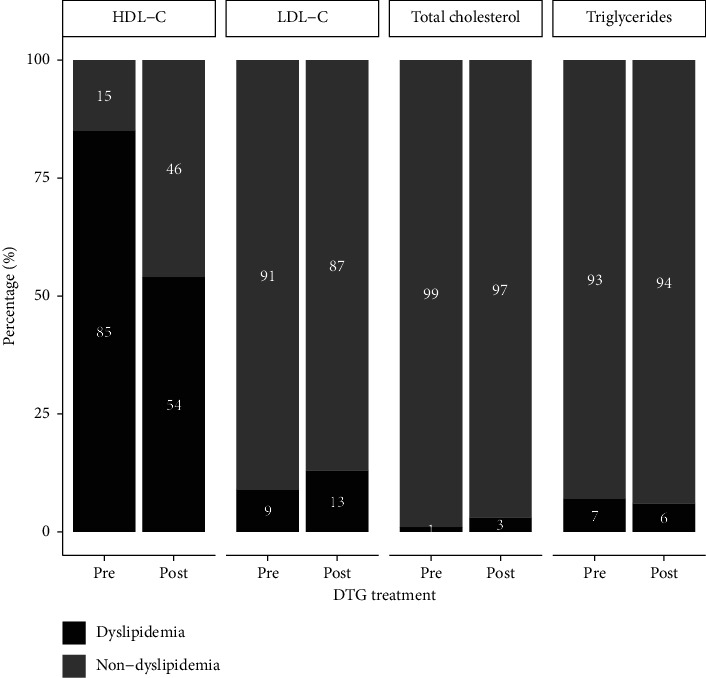
Bar graphs showing the prevalence of dyslipidemia and non-dyslipidemia between pre- and post-Dolutegravir (DTG) treatment. Dyslipidemia was defined as total cholesterol > 5.18 mmol/L, LDL-C >2.59 mmol/L, HDL-C <1.04 mmol/L and triglycerides > 2.26 mmol/L [12].

As shown in Figure 1, the predominant dyslipidemia at baseline among study participants was characterised by low HDL-C, affecting 85% of the cohort. After 6 month on a DTG-based ART regimen, there was a statistically significant increase in mean HDL-C levels, resulting in a notable 31% reduction in the overall prevalence of dyslipidemia. Conversely, the prevalence of elevated LDL-C concentration observed a modest 4% increment post-DTG, relative to the baseline value. Additionally, marginal changes were noted in the prevalence of hypertriglyceridemia (1%) and hypercholesterolemia (2%) during the same period.

## 4. Discussion

In the present study conducted in Harare, Zimbabwe, we examined alterations in weight and lipid levels among individuals taking DTG-based regimen ART for 6 month. Our findings revealed noteworthy increases in body weight, BMI, TC and HDL-C levels, coupled with a significant decrease in TG levels. Furthermore, our findings highlighted a reduction in the prevalence of dyslipidemia following DTG treatment, as evidenced by lowered TG levels and increased HDL-C levels.

We found significant increases in weight and BMI among individuals following 6-month post-DTG initiation. The mean weight among participants increased to 67.6 kgs from 61.8 kgs at baseline (*p*=0.001), with an average weight gain of 5.8 kg (*p*=0.001). The mean BMI increased to 24.3 kg/m^2^ from 22.1 kg/m^2^ (*p*=0.005). Our observations are consistent with previous studies that have shown an association between the use of INSTIs and gains in body weight and BMI [13–15]. Similarly, a study conducted among adult, treatment-naïve individuals living with HIV in the United States demonstrated that, following 18-month post-ART, participants on DTG gained 6.0 kg, compared to 2.6 kg for NNRTIs (*p* < 0.05) [15].

In addition, our findings are also comparable to two large randomised clinical trials conducted in Cameroon (NAMSAL study) and South Africa (ADVANCE study) demonstrated that the DTG-based regimen was associated with greater weight gain than in the group receiving an efavirenz-based ART regimen [16–18]. In the NAMSAL study, weight gain was greater in the DTG group (median weight gain, 5.0 kg in the DTG group and 3.0 kg in the efavirenz 400 mg group, *p* < 0.001, and incidence of obesity, 22% in the DTG group and 16% in the efavirenz 400 mg group, *p*=0.043). In South Africa, the advance study reported a mean weight gain of 8.9 kg in the TAF/FTC + DTG arm, 5.9 kg in the TDF/FTC + DTG arm, and 3.2 kg in the TDF/FTC/EFV arm and was greatest among women, those taking TAF, and those with lower baseline CD4 counts. These observations were made after 48, 96 and 192 weeks of ART, thus demonstrating DTG-associated weight gain both in the short- and long-term [16–18]. The phenomenon of weight gain associated with DTG is consistently observed across diverse socioeconomic statuses, healthcare infrastructures, and genetic backgrounds.

While weight gain may be common after initiating ART, the increase associated with INSTIs appears to be beyond typical recovery-related gains [19]. Studies have demonstrated that the mechanisms behind these changes may be multiple. In one experimental study in mice, DTG was shown to target mitochondria in brown adipocytes [8]. The study also showed that this disrupted the mitochondrial thermogenic function which then suppressed energy expenditure and thereby contributing to weight gain [8]. Another hypothesis tested in vitro showed that DTG interferes with the melanocortin signalling system and subsequently increases appetite and thus weight gain [20]. Studies have also shown that ART is associated with reversing the catabolic state that is characteristic of malnourished patients and inflammation [21], thus DTG action was associated with increased appetite and nutrient absorption, and therefore weight gain. These hypotheses underscore the multifaceted nature of weight dynamics in the context of ART, with potential involvement of both viral load-related factors and hormonal modulation.

Our findings on increasing weight are a cause for concern on the contribution of DTG-based regimens to the prevalence of overweight and obesity. These risk factors have been associated with insulin resistance, diabetes mellitus, hypertension, coronary artery disease, lipodystrophy and dyslipidemia [1, 2]. This understanding therefore should raise awareness on the need to monitor weight and BMI and identify risk factors associated with weight changes in patients on DTG-based ART regimens to minimise associated risk of noncommunicable diseases. In the present study, current employment was also a risk factor associated with weight gain (OR = 3.69, 95% CI 1.46–9.32, *p*=0.006). We hypothesise that the sedentary nature in modern occupations (as was the case in our urban sample) and long working hours could account for this association. It has been previously reported that sedentary lifestyles are associated with reduced physical activity, and unhealthy diet which in turn cause weight gain [22]. Furthermore, the findings on increased rate of change in weight gain highlight the critical role of ART in enhancing CD4 cell counts and emphasise the necessity of identifying lifestyle modifications to counteract the increase in lipid levels associated with older age.

Our findings on the favourable lipid levels after 6 month on the DTG-based ART from their baseline values are consistent with those shown in other studies. Thus, in the RESPOND study which was conducted in 4577 participants from Australia and Europe, 2 years of DTG-based ART was associated with a lower incidence of dyslipidemia than with protease-inhibitor containing regimens [23]. Similarly, a review on RCTs by Saumoy et al. showed that DTG had superior lipid profiles than efavirenz or protease inhibitor boosted regimens [24]. From Uganda, Kigongo et al. demonstrated a high prevalence of low HDL-C (72.1%, *n* = 341) but a high burden of dyslipidemia among participants who had been exposed to DTG-based regimens for approximately 35 months [25]. In contrast, the NAMSAL study conducted in Cameroon reported that TC increased from baseline values at 48 weeks after being on a DTG-based regimen [26]. However, when compared to the other treatment arm which was based on efavirenz, there were no differences in the changes to TC and TG levels with those observed in the DTG group [26]. In Ethiopia, individuals receiving efavirenz had higher TC and HDL-C levels than those on DTG despite similarities in the prevalence of dyslipidemia between the two groups (75.0% vs. 79.7%) [27]. Together with our study, emerging evidence is therefore showing favourable lipid profiles associated with DTG-based therapy which is important for gaining insight into these new treatment approaches. However, because DTG-based therapy has only been introduced in the last 5 years in our population, more studies on further exploration and monitoring on its use and associated effects are warranted.

We acknowledge our study had some limitations. Firstly, we did not include a comparison group and were therefore unable to make clear inferences on the causality between DTG and the increase in weight, BMI and dyslipidemia. Furthermore, we did not collect baseline data on other possible underlying secondary causes of dyslipidemia such as hyperthyroidism, Cushing's syndrome, chronic kidney disease and diabetes mellitus. Thirdly, we did not collect data on the use of lipid-lowering therapy including statins or other medications that could have affected lipid metabolism. Lastly, we had a small sample size and the limited follow-up period that curtailed longer-term assessment of the extent to which weight and lipid levels may change following DTG initiation. However, understanding the nature of the association between any ART and weight gain is complex and requires factoring in confounders such as concomitant medications, lifestyle, return-to-health phenomenon and possible effect of prior regimens on weight. Despite these limitations, our study had some strengths, which include its prospective cohort nature and the measurement of lipid levels using standardised assays.

## 5. Conclusions

Upon the initiation of DTG, discernible elevations were observed in body weight, BMI and lipid levels. These findings, while indicative of short-term effects, underscore the imperative for further investigative efforts aimed at elucidating the prolonged consequences associated with DTG-induced weight gain and increased lipid levels and its underlying mechanisms.

## Figures and Tables

**Table 1:** Baseline sociodemographic characteristics of study population.

Variable	Total *N* = 130	Males *N* = 56	Females *N* = 74	*p* value
Age (years)	40 ± 11	41 ± 10	39 ± 12	0.376
Weight (kg)	61.9 ± 12.6	61.7 ± 10.5	62 ± 14.1	0.885
BMI (kg/m^2^)	22 (19.2–26)	20.3 (18.5–23.5)	23.2 (20.4–26.9)	**0.003**
ART status, *n* (%)				
Naïve	107 (82)	45 (80)	62 (84)	0.612
Defaulters	23 (18)	11 (20)	12 (16)	
CD4 cell count (cells/mm^3^)	196 (98–357)	191.5 (76.5–318.5)	213 (109–362)	0.493
Marital status, *n* (%)				
Married	72 (55.4)	32 (57.14)	40 (54.05)	0.726
Not married	58 (44.6)	24 (42.86)	34 (45.95)	
Education, *n* (%)				
Primary	7 (5.38)	1 (1.79)	6 (8.11)	0.372
Secondary	107 (82.31)	48 (85.71)	59 (79.73)	
Tertiary	16 (12.31)	7 (12.50)	9 (12.16)	
Employment, *n* (%)				
Employed	61 (46.92)	32 (57.14)	29 (39.19)	**0.042**
Unemployed	69 (53.08)	24 (42.86)	45 (60.81)	

*Note:* Data expressed as mean ± SD, median (IQR) and frequency (proportion). Nonmarried was defined as either Single (*n* = 25), divorced (*n* = 21) or widowed (*n* = 12). Bold values represent *p* value statistically significant (*p* < 0.05).

Abbreviations: ART, antiretroviral therapy; BMI, body mass index; IQR, interquartile range; SD, standard deviation.

**Table 2:** Comparison of weight, BMI and lipid levels before and after DTG-based ART (*n* = 105).

Variables	Baseline (pre-DTG)	6 months (post-DTG)	*p* value
Weight (kg)	61.8 (11.5)	67.6 (13.1)	0.001
BMI (kg/m^2^)	22.1 (19.3–26.1)	24.3 (21.1–26.7)	0.005
TG (mmol/L)	1.10 (0.86–1.66)	0.80 (0.61–1.20)	< 0.001
TC (mmol/L)	2.81 (2.29–3.34)	3.10 (2.60–3.79)	0.003
HDL-C (mmol/L)	0.71 (0.55–0.93)	0.98 (0.81–1.25)	< 0.001
LDL-C (mmol/L)	1.49 (1.09–2.11)	1.70 (1.23–2.15)	0.171

*Note:* Data expressed as mean (SD), median (IQR) and frequency (proportion).

Abbreviations: BMI, body mass index; DTG, dolutegravir; HDL-C, high-density lipoprotein cholesterol; IQR, interquartile range; LDL, low-density lipoprotein cholesterol; TC, total cholesterol; TG, triglyceride.

**Table 3:** Univariate and multivariable logistic regression on the relationship between weight gain and associated baseline determinants.

Variables	Univariate				Multivariate		
	Proportion	OR	95% CI	*p* value	OR	95% CI	*p* value
Age, in years							
< 40	0.52	1.15	0.52–2.57	0.726			
≥ 40	0.48						
Sex							
Male	0.43	0.71	0.31–1.60	0.410			
Female	0.57						
Baseline CD4, cell / mm^3^							
< 200	0.50	1.23	0.54–2.78	0.628			
≥ 200	0.50						
Marital status							
Married	0.55	0.62	0.27–1.39	0.66			
Not married	0.45						
Employment							
Unemployed	0.53	2.81	1.23–6.45	**0.014**			
Employed	0.47				3.69	1.46–9.32	**0.006**

*Note:* Bold values represent *p* value statistically significant (*p* < 0.05).

Abbreviations: CI, confidence interval; OR, odds ratio.

## Data Availability

De-identified datasets used and/or analysed during the current study are available from the corresponding author on reasonable request.

## Nomenclature

WHO: World Health Organization
DTG: Dolutegravir
BMI: Body mass index
PLWH: People living with HIV
ART: Antiretroviral therapy
HDL-C: High-density lipoprotein cholesterol
TC: Total cholesterol
TG: Triglycerides
LDL-C: Low-density lipoprotein cholesterol
ZIMPHIA: Zimbabwe population-based HIV impact assessment
INSTI: Integrase strand transfer inhibitor
PHFCC: Parirenyatwa Hospital Family Care Centre
EDTA: Ethylenediaminetetraacetic acid
SD: Standard deviation
OR: Odds ratio
IQR: Interquartile range
NCEPATP III: National cholesterol education programme, adult treatment panel

## Supporting Information

Supporting Information: Table 1 shows baseline characteristics between ART naïve individuals and those who had previously defaulted. Table 2 shows the comparison of baseline characteristics between retained and loss to follow-up. Table 3 shows factors associated with an increase in HDL-C levels. Table 4 shows factors associated with an increase in TC levels. Table 5 shows factors associated with an increase in TG levels. Table 6 shows univariate and multivariable linear regression on baseline factors associated with the rate of change in weight over the 6-month study period. Table 7 shows univariate and multivariable linear regression on baseline factors associated with the rate of change in total cholesterol over the 6-month study period. Table 8 shows univariate and multivariable linear regression on baseline factors associated with the rate of change in LDL-cholesterol over the 6-month study period. Table 9 shows univariate and multivariable linear regression on baseline factors associated with the rate of change in triglycerides over the 6-month study period. Table 10 shows univariate and multivariable linear regression on baseline factors associated with the rate of change in HDL-cholesterol over the 6-month study period.
