# Supplementary material for: Weight and Lipid Levels in People Living With HIV and Initiating a Dolutegravir-Based Regimen in a Resource Limited Setting: A Prospective Study
Source: AIDS Res Treat. 2024 Nov 14;2024:4620951. doi: 10.1155/2024/4620951 (PMC11581792; doi:10.1155/2024/4620951)
Supplement: Supporting Information — Additional supporting information can be found online in the Supporting Information section. [file 4620951.f1.docx]

**SUPPLEMENTARY MATERIAL**

**Supplementary Table 1**: Baseline characteristics between ART naïve individuals and those who had previously defaulted

| Variables | ART naïve (n=107) | Previously defaulted (n=23) | P value |
| --- | --- | --- | --- |
| Age, years | 39.87 ± 11.51 | 37.86 ± 10.82 | 0.45 |
| Weight, kg | 60 (52 – 72) | 60 (55 – 66) | 0.92 |
| BMI, kg/m^2^ | 21.75 (19.05 – 26.15) | 22.15 (20.00 – 24.65) | 0.83 |
| CD4, cells/mm^3^ | 169 (74 – 351) | 275 (198 – 436) | 0.04 |
| Married | 64 (59.81) | 8 (34.78) | 0.03 |
| Employed | 53 (49.53) | 8 (34.78) | 0.20 |
| TC (mmol/L) | 2.87 (2.20 – 3.43) | 2.83 (2.36 – 3.24) | 0.70 |
| HDL-C (mmol/L) | 0.73 (0.53 – 0.93) | 0.75 (0.62 – 0.92) | 0.65 |
| LDL-C (mmol/L) | 1.60 (1.05 – 2.21) | 1.30 (1.15 – 1.79) | 0.26 |
| Triglycerides (mmol/L) | 1.09 (0.86 – 1.63) | 1.14 (0.86 – 1.71) | 0.54 |

Data expressed as mean (SD), median (IQR) and frequency (proportion). IQR=Interquartile range, TG=Triglyceride, TC=Total cholesterol, HDL-C=High density lipoprotein-cholesterol, LDL=Low density lipoprotein-cholesterol. Level of significance after Bonferroni correction, p<0.005

**Supplementary Table 2**: Comparison of baseline characteristics between retained and loss to follow-up

| Variables | Retained | Loss to follow-up | P value |
| --- | --- | --- | --- |
| Age (years) | 39.88 ± 12.02 | 38.41 ± 9.21 | 0.52 |
| Female | 56 (57.14) | 18 (56.3) | 0.93 |
| Weight (kg) | 61.78 ± 11.69 | 62.07 ± 15.31 | 0.91 |
| CD4 (cell /mm^3^) | 192 (98 – 328) | 233.5 (77 – 423) | 0.92 |
| Married | 54 (55.10) | 18 (56.3) | 0.38 |
| Employed | 42 (42.85) | 19 (59.38) | 0.39 |
| TC (mmol/L) | 2.79 (2.29 – 3.34) | 3.11 (2.16 – 3.58) | 0.83 |
| HDL-C (mmol/L) | 0.72 (0.55 – 0.92) | 0.75 (0.52 – 0.94) | 0.78 |
| LDL-C (mmol/L) | 1.50 (1.07 – 2.08) | 1.66 (0.90 – 2.14) | 0.89 |
| Triglycerides (mmol/L) | 1.08 (0.86 – 1.65) | 1.29 (0.79 – 1.70) | 0.77 |

Data expressed as mean (SD), median (IQR) and frequency (proportion). IQR=Interquartile range, TG=Triglyceride, TC=Total cholesterol, HDL-C=High density lipoprotein-cholesterol, LDL=Low density lipoprotein-cholesterol.

**Supplementary Table 3**: Factors associated with an increase in HDL-C levels

| **Variables** | **Unadjusted** | | | | **Adjusted** | | |
| --- | --- | --- | --- | --- | --- | --- | --- |
|  | Proportion | OR | **95% CI** | P value | OR | 95%CI | P value |
| **Age (n=130), years**  <40  ≥40 | 0.52  0.48 | 1.00 | 0.45-2.21 | 1.00 | 1.42 | 0.54-3.74 | 0.476 |
| **BMI (n=100), kg/m^2^**  <30  ≥30 | 0.93  0.07 | 2.10 | 0.36-12.14 | 0.406 | 3.55 | 0.36-35.15 | 0.279 |
| **Sex (n=130)**  Male  Female | 0.43  0.57 | 1.09 | 0.49-2.41 | 0.839 | 0.75 | 0.28-1.99 | 0.559 |
| **Baseline CD4 (n=121), cell /mm^3^**  <200  ≥200 | 0.50  0.50 | 1.76 | 0.77-4.00 | 0.178 | 1.90 | 0.72-5.03 | 0.198 |
| **Marital Status (n=130)**  Married  Not married | 0.55  0.45 | 1.39 | 0.63-3.09 | 0.417 | 1.58 | 0.61-4.05 | 0.345 |
| **Employment (n=130)**  Unemployed  Employed | 0.53  0.47 | 1.52 | 0.68- 3.38 | 0.310 | 1.85 | 0.72-4.75 | 0.199 |

BMI=Body mass index, OR=Odds ratio, CI=Confidence interval

**Supplementary Table 4**: Factors associated with an increase in TC levels

| **Variables** | **Unadjusted** | | | | **Adjusted** | | |
| --- | --- | --- | --- | --- | --- | --- | --- |
|  | Proportion | OR | **95% CI** | P value | OR | 95%CI | P value |
| **Age (n=130), years**  <40  ≥40 | 0.52  0.48 | 2.29 | 1.02-5.14 | **0.045** | 3.37 | 1.20-9.50 | **0.022** |
| **BMI(n=100) kgs/m^2^**  <30  ≥30 | 0.93  0.07 | 2.21 | 0.38-12.76 | 0.375 | 3.11 | 0.27-35.90 | 0.362 |
| **Sex (n=130)**  Male  Female | 0.43  0.57 | 0.47 | 0.21-1-06 | 0.069 | 0.36 | 0.13-1.02 | 0.055 |
| **Baseline CD4 (n= 121), cell /mm^3^**  <200  ≥200 | 0.50  0.50 | 1.48 | 0.65-3.35 | 0.349 | 1.49 | 0.54-4.12 | 0.439 |
| **Marital Status (n=130)**  Married  Not married | 0.55  0.45 | 1,00 | 0.45-2,22 | 1.000 | 1.10 | 0.41-2.94 | 0.855 |
| **Employment (n=130)**  Unemployed  Employed | 0.53  0.47 | 0.66 | 0.30-1.47 | 0.310 | 0.80 | 0.30-2.10 | 0.650 |

BMI=Body mass index, OR=Odds ratio, CI=Confidence interval

**Supplementary Table 5**: Factors associated with an increase in TG levels

| **Variables** | **Unadjusted** | | | | **Adjusted** | | |
| --- | --- | --- | --- | --- | --- | --- | --- |
|  | Proportion | OR | **95% CI** | P | OR | 95%CI | p |
| **Age (n=130), years**  <40  ≥40 | 0.52  0.48 | 2.29 | 1.02-5.14 | **0.045** | 1.50 | 0.59-3.82 | 0.399 |
| **BMI(n=100) kgs/m^2^**  <30  ≥30 | 0.93  0.07 | 0.5 | 0.09-2.89 | 0.438 | 0.64 | 0.09-4.35 | 0.648 |
| **Sex (n=130)**  Male  Female | 0.43  0.57 | 1.28 | 0.58-2.85 | 0.542 | 0.98 | 0.37-2.59 | 0.960 |
| **Baseline CD4 (n= 121), cell /mm^3^**  <200  ≥200 | 0.50  0.50 | 0.57 | 0.25-1.29 | 0.178 | 0.50 | 0.19-1.29 | 0.151 |
| **Marital Status (n=130)**  Married  Non-married | 0.55  0.45 | 0.85 | 0.38-1.88 | 0.685 | 0.89 | 0.35-2.26 | 0.801 |
| **Employment (n=130)**  Unemployed  Employed | 0.53  0.47 | 1.09 | 0.49-2.41 | 0.839 | 1.16 | 0.46-2.95 | 0.747 |

BMI=Body mass index, OR=Odds ratio, CI=Confidence interval

**Supplementary Table 6**: Univariate and multivariable linear regression on baseline factors associated with the rate of change in weight over the 6-month study period

| Variables | Unadjusted | | | | Adjusted | | |
| --- | --- | --- | --- | --- | --- | --- | --- |
|  | Proportion | Beta | 95% CI | P value | Beta | 95% CI | P value |
| Age, years  <40  ≥40 | 0.52  0.48 | -0.33 | -0.88 – 0.20 | 0.22 | - | - | - |
| Sex  Male  Female | 0.43  0.57 | -0.03 | -0.59 – 0.52 | 0.90 | - | - | - |
| Baseline CD4  <200 cells/mm^3^  ≥200 cells/mm^3^ | 0.50  0.50 | 0.84 | 0.32 – 1.36 | 0.002 | 0.82 | 0.31 – 1.34 | 0.002 |
| Marital status  Married  Not Married | 0.55  0.45 | -0.02 | -0.57 – 0.53 | 0.93 | - | - | - |
| Employment  Employed  Unemployed | 0.53  0.47 | 0.53 | -0.01 – 1.07 | 0.06 | 0.50 | -0.01 – 1.07 | 0.06 |

Independent variable: rate of change in weight in 6-months = change in weight /6. Dependent variables age, sex, CD4 count, marital and employment status.

**Supplementary Table 7**: Univariate and multivariable linear regression on baseline factors associated with the rate of change in total cholesterol over the 6-month study period

| Variables | Unadjusted | | | | Adjusted | | |
| --- | --- | --- | --- | --- | --- | --- | --- |
|  | Proportion | Beta | 95% CI | P value | Beta | 95% CI | P value |
| Age, years  <40  ≥40 | 0.52  0.48 | **-0.07** | **-0.13 – -0.02** | **0.01** | - | - | - |
| Sex  Male  Female | 0.43  0.57 | 0.03 | -0.02 – 0.10 | 0.22 | - | - | - |
| Baseline CD4  <200 cells/mm^3^  ≥200 cells/mm^3^ | 0.50  0.50 | -0.01 | -0.07 – 0.05 | 0.66 |  |  |  |
| Marital status  Married  Not Married | 0.55  0.45 | 0.02 | -0.04 – 0.08 | 0.46 | - | - | - |
| Employment  Employed  Unemployed | 0.53  0.47 | 0.02 | -0.04 – 0.08 | 0.57 |  |  |  |

Independent variable: rate of change in total cholesterol in 6-months = change in total cholesterol/6. Dependent variables age, sex, CD4 count, marital and employment status.

**Supplementary Table 8:** Univariate and multivariable linear regression on baseline factors associated with the rate of change in LDL-cholesterol over the 6-month study period

| Variables | Unadjusted | | | | Adjusted | | |
| --- | --- | --- | --- | --- | --- | --- | --- |
|  | Proportion | Beta | 95% CI | P value | Beta | 95% CI | P value |
| Age, years  <40  ≥40 | 0.52  0.48 | -0.06 | -0.11 – -0.01 | 0.02 | **-0.06** | **-0.12 – -0.01** | 0.02 |
| Sex  Male  Female | 0.43  0.57 | <-0.01 | -0.05 – 0.05 | 0.99 | - | - | - |
| Baseline CD4  <200 cells/mm^3^  ≥200 cells/mm^3^ | 0.50  0.50 | -0.02 | -0.08 – 0.03 | 0.45 | - | - | - |
| Marital status  Married  Not Married | 0.55  0.45 | -.03 | -0.09 – 0.02 | 0.16 | -0.04 | -0.09 – 0.01 | 0.14 |
| Employment  Employed  Unemployed | 0.53  0.47 | .004 | -0.05 – 0.05 | 0.88 | - | - | - |

Independent variable: rate of change in LDL-cholesterol in 6-months = change in LDL-cholesterol/6. Dependent variables age, sex, CD4 count, marital and employment status.

**Supplementary Table 9:** Univariate and multivariable linear regression on baseline factors associated with the rate of change in triglycerides over the 6-month study period

| Variables | Unadjusted | | | | Adjusted | | |
| --- | --- | --- | --- | --- | --- | --- | --- |
|  | Proportion | Beta | 95% CI | P value | Beta | 95% CI | P value |
| Age, years  <40  ≥40 | 0.52  0.48 | -0.03 | -0.08 – 0.01 | 0.16 | -0.03 | -0.08 – 0.01 | 0.16 |
| Sex  Male  Female | 0.43  0.57 | 0.02 | -0.03 – 0.07 | 0.36 | - | - | - |
| Baseline CD4  <200 cells/mm^3^  ≥200 cells/mm^3^ | 0.50  0.50 | -0.01 | -0.06 – 0.04 | 0.73 | - | - | - |
| Marital status  Married  Not Married | 0.55  0.45 | 0.04 | -0.01 – 0.09 | 0.11 | 0.04 | -0.01 – 0.09 | 0.12 |
| Employment  Employed  Unemployed | 0.53  0.47 | 0.02 | -0.03 – 0.07 | 0.48 | - | - | - |

Independent variable: rate of change in triglycerides in 6-months = change in triglycerides/6. Dependent variables age, sex, CD4 count, marital and employment status.

**Supplementary Table 10**: Univariate and multivariable linear regression on baseline factors associated with the rate of change in HDL-cholesterol over the 6-month study period

| Variables | Unadjusted | | | | Adjusted | | |
| --- | --- | --- | --- | --- | --- | --- | --- |
|  | Proportion | Beta | 95% CI | P value | Beta | 95% CI | P value |
| Age, years  <40  ≥40 | 0.52  0.48 | 0.02 | -0.40 – 0.46 | 0.90 | - | - | - |
| Sex  Male  Female | 0.43  0.57 | 0.37 | -0.06 – 0.79 | 0.09 |  |  |  |
| Baseline CD4  <200 cells/mm^3^  ≥200 cells/mm^3^ | 0.50  0.50 | -0.01 | -0.44 – 0.42 | 0.95 | - | - | - |
| Marital status  Married  Not Married | 0.55  0.45 | -0.09 | -0.53 – 0.33 | 0.66 | - | - | - |
| Employment  Employed  Unemployed | 0.53  0.47 | 0.24 | -0.19 – 0.67 | 0.27 | - | - | - |

Independent variable: rate of change in HDL-cholesterol in 6-months = change in HDL-cholesterol/6. Dependent variables age, sex, CD4 count, marital and employment status.
